# *Lactococcus lactis* subsp. Cremoris reprograms systemic metabolism and protects against myocardial injury

**DOI:** 10.1080/19490976.2025.2609426

**Published:** 2026-01-04

**Authors:** C. Anthony Gacasan, Crystal R. Naudin, Jaclyn Weinberg, Lauren C. Askew, Maria E. Barbian, Dean P. Jones, Rheinallt M. Jones

**Affiliations:** a Division of Gastroenterology, Hepatology, and Nutrition, Department of Pediatrics, Emory University School of Medicine, Atlanta, GA, USA; b Division of Pulmonary, Allergy, Critical Care and Sleep Medicine, Department of Medicine, Emory University School of Medicine, Atlanta, GA, USA; c Division of Neonatology, Department of Pediatrics, Emory University School of Medicine, Atlanta, GA, USA

**Keywords:** Gut-heart axis, *Lactococcus lactis* subsp. Cremoris, probiotic, atherosclerosis, myocardial infarction, metabolomics

## Abstract

Therapeutic microbes are increasingly recognized as potent modulators of host physiology, yet their influence beyond the gut remains underexplored. While *Lactococcus lactis* subsp. Cremoris (LLC) has been shown to preserve gut epithelial integrity and counteract Western diet-induced metabolic syndrome in murine models, its effects on extraintestinal systems such as the gut-cardiovascular axis, are not well defined. In this study, we employed a multimodal experimental approach to investigate whether LLC confers cardioprotective benefits. We showed that LLC supplementation significantly preserved cardiac function and reduced myocardial scarring following ischemia-reperfusion injury. Untargeted metabolomic profiling of cardiac tissue revealed distinct shifts in the cardiac metabolome, with pathway enrichment analyses highlighting alterations in glutathione metabolism, fatty acid degradation, and other key cardiometabolic pathways. Furthermore, we employed weighted gene coexpression network analysis of our cardiac metabolomics dataset to capture the system-level changes induced by LLC. These findings position LLC as a promising probiotic capable of promoting systemic metabolic reprogramming and mitigating adverse cardiovascular outcomes. Our data support a model in which LLC exerts cardioprotective effects through the modulation of lipid metabolism and enhancement of anti-inflammatory signaling along the gut‒heart axis.

## Introduction

Cardiovascular diseases (CVD) remain the leading cause of morbidity and mortality worldwide, with the prevalence of coronary heart disease and heart failure projected to increase by more than 40% by 2035.^1^ This escalating burden is compounded by the widespread incidence of comorbid conditions such as obesity and metabolic syndrome, which significantly elevate CVD risk and place increasing strain on global healthcare systems. Myocardial infarction (MI) is a primary contributor to heart failure, resulting in the death of cardiomyocytes due to ischemic injury.^2^ In adult mammals, the heart exhibits limited regenerative capacity, and the damaged myocardium is typically replaced by fibrotic scar tissue. This maladaptive remodeling process compromises cardiac structure and function, ultimately leading to progressive contractile dysfunction and heart failure.^3^ Given the limited intrinsic repair mechanisms of the adult heart, there is an urgent need for innovative therapeutic strategies that can minimize postinfarction scarring and preserve myocardial function.

Recent evidence suggests that the gut microbiome plays a pivotal role in cardiovascular health, with growing interest in the potential of probiotics, which are defined as beneficial microbes capable of modulating host physiology to influence disease outcomes.^4-6^ While the effects of probiotics on intestinal health are well characterized, their systemic impacts, particularly on the gut‒cardiovascular axis, remain underexplored. Notably, probiotic interventions have been shown to ameliorate metabolic comorbidities such as diet-induced obesity and hepatic steatosis, both of which are major contributors to CVD risk.^7-12^ These findings underscore the potential of microbiome-based therapies to improve vascular health and metabolic homeostasis. Despite this promise, the capacity of probiotics to directly influence cardiac injury and post-MI remodeling remains poorly understood. Addressing this gap could reveal novel mechanisms of cardioprotection and inform the development of microbiome-targeted interventions for CVD.

Using a high-throughput screening platform designed to identify probiotics with enhanced therapeutic potential, we previously identified *Lactococcus lactis* subsp. Cremoris ATCC19257 (LLC) as a potent strain capable of suppressing inflammation and promoting epithelial repair in models of acute and chronic intestinal injury.^13^^,^^14^ Mechanistically, LLC activates the nuclear factor erythroid 2-related factor 2 (Nrf2) pathway, driving cytoprotective and antioxidant responses in the gut.^14^^,^^15^ In the context of Western-style diet (WSD) feeding, LLC has demonstrated efficacy in mitigating features of metabolic syndrome, including reduced weight gain, preserved glucose tolerance, decreased hepatic steatosis, and favorable shifts in lipid metabolism.^16^ Importantly, LLC consistently outperformed the widely studied probiotic *Lactobacillus rhamnosus* GG (LGG), which failed to confer protection in parallel assays.^16^ These findings position LLC as a promising next-generation probiotic with systemic therapeutic potential. However, its effects on cardiovascular outcomes, particularly in the context of myocardial injury and atherosclerosis, have not been investigated.

In this study, we demonstrate that supplementation with LLC elicits robust cardioprotective effects in preclinical models. To investigate the underlying metabolic mechanisms, we performed untargeted metabolomic profiling of cardiac tissue using liquid chromatography–mass spectrometry (LC-MS). Comparative analyzes of mice treated with LLC, LGG, or vehicle revealed distinct shifts in the cardiac metabolome. Pathway enrichment analyzes^17^ identified significant alterations in glutathione metabolism, fatty acid degradation, and other key cardiometabolic processes. Furthermore, we employed weighted gene coexpression network analysis (WGCNA) on our cardiac metabolomics dataset, a tool commonly used in microarray and RNA sequencing that can be used to identify coregulated networks of metabolites to better capture the system-level changes induced by LLC.^18-21^ This analysis revealed that LLC supplementation induces comprehensive reprogramming of cardiometabolic networks rather than isolated shifts in individual metabolites. Notably, LLC enhances pathways associated with antioxidant defense, fatty acid utilization, and amino acid metabolism. In line with this systems-level remodeling, interrogation of the curated Microbial Metabolite Database (MiMeDB)^22^ revealed that LLC supplementation reshaped the cardiac pool of gut-associated metabolites, enriching niacinamide, inosine, and hypoxanthine, while depleting spermine and acyl-carnitines. These changes underscore the intimate connection between gut microbial activity and cardiac metabolic health. Collectively, our findings demonstrate that LLC supplementation mitigates cardiac injury and suggest that gut microbiota-driven metabolic modulation may underlie its protective effects.

## Results

### Supplementation with *Lactococcus lactis* subsp. Cremoris protects mice against ischemia-reperfusion injury

**Figure 1.**
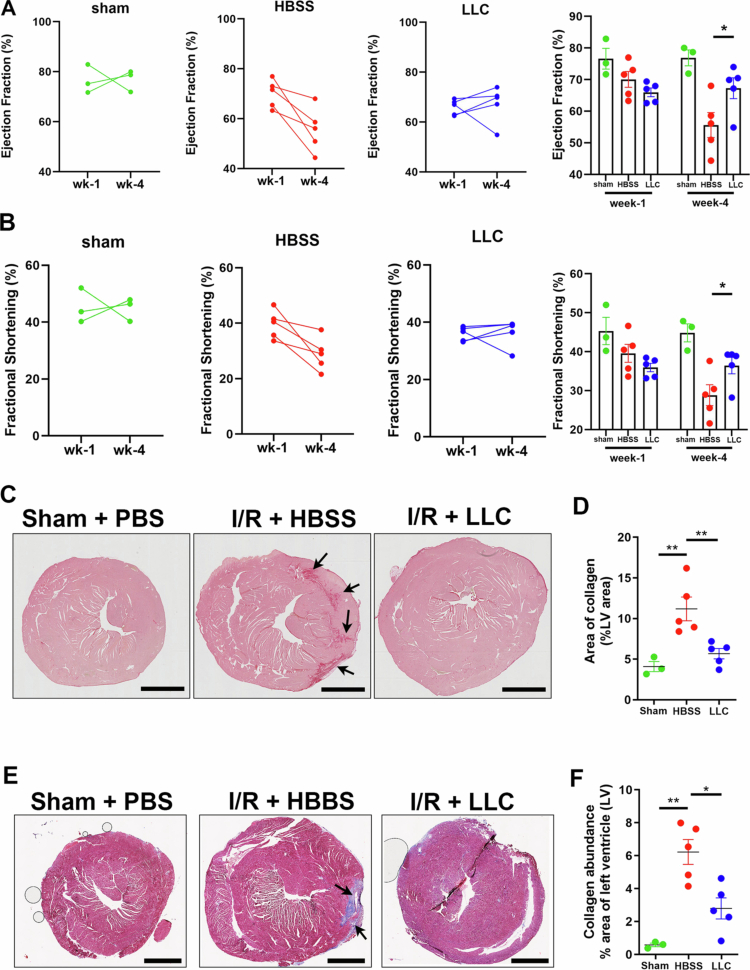
*L. lactis* improves cardiac function and reduces scarring after MI. (A) Echocardiography at 1 and 4 weeks post-MI for the assessment of heart function by the ejection fraction in LLC- or HBSS-treated mice. (B) Echocardiography at 1 and 4 weeks post-MI for the assessment of heart function by fractional shortening in LLC- or HBSS-treated mice. (C) Histological assessment of cardiac sections stained with Sirius Red to evaluate tissue collagen deposition in the mice outlined in (A). White arrow points to Sirius Red-positive tissue. (D) Quantification of Sirius Red-positive tissue in samples outlined in (C). (E) Histological assessment of cardiac sections stained with Masson's trichome sections were stained with Masson's trichrome to evaluate myocardial architecture, fibrosis, and collagen deposition. (F) Quantification of Masson's trichrome-positive tissue in the samples described in (E). Statistical significance was determined via one-way ANOVA followed by Tukey's post hoc test. The values are as follows: mean ± SEM, *n* = 3 for the sham group and *n* = 5 for the HBSS- and LLC-supplemented groups; *P < .05, **P < .01.

We evaluated the cardioprotective effects of LLC supplementation in a murine model of myocardial ischemia-reperfusion (I/R) injury. The mice were administered daily oral doses of LLC (2 × 10^9^ CFU) or Hanks' balanced salt solution (HBSS) vehicle control for two weeks prior to the induction of MI via a 30-minute ligation of the left coronary artery. Following a 3-d postoperative recovery period, LLC and HBSS supplementation was resumed, and cardiac function was evaluated by parasternal long axis (PSLAX) echocardiography at one and four weeks following ischemia-reperfusion injury (Supplementary Figure 1). One week following I/R injury, the ejection fraction (EF) values remained above 50%, and the fractional shortening (FS) values were above 30% across all the experimental groups. However, by four weeks post-I/R, EF declined to approximately 50%, and FS decreased to approximately 30% in the control HBSS-treated mice, which is consistent with progressive cardiac dysfunction. (Figure 1A and B) In contrast, mice treated with LLC exhibited preserved cardiac function with 4 out of 5 LLC-treated I/R mice maintaining EF values above 50%, with a trend toward improved EF between weeks 1 and 4. Similarly, FS values remained above 30% in 4 out of 5 LLC-treated I/R model mice. (Figure 1A and B). Despite these protective effects, both I/R groups demonstrated EF and FS values that were lower than those observed in sham-operated controls, underscoring the severity and extent of the injury and the partial nature of the functional rescue (Figure 1A and B). Histological analysis revealed a marked reduction in cardiac scar size in the LLC-treated group, as determined by analysis of collagen abundance (Figure 1C and D) and fibrosis (Figure 1E and F). These data indicate that LLC supplementation elicits myocardial preservation following I/R injury.

### LLC activates Nrf2 signaling in cardiac tissue

**Figure 2.**
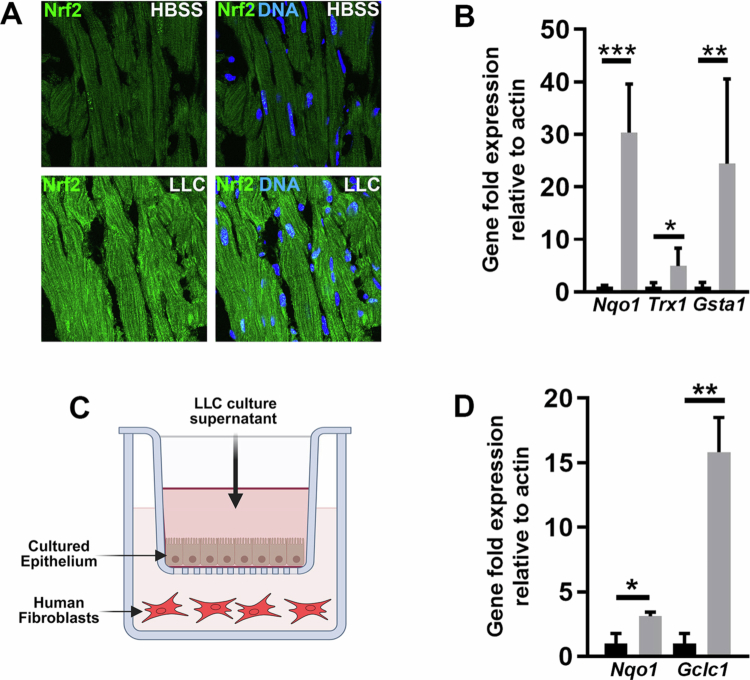
LLC activates Nrf2 signaling in cardiac tissue. (A) Immunofluorescence for the abundance of Nrf2 in cardiac tissue of germ-free mice treated with 2 × 10^9^CFU of LLC, or vehicle control for 4 hours. (B) qPCR analysis of cardiac tissue from (A) for the detection of transcript enrichment of Nqo1, Trx1 and GstA1. (C) Diagram of trans-well experimental setup that includes SK-CO15 cells human colon cell line within the insert, and human cardiac fibroblasts propagated in the main well. Filter sterilized LLC-conditioned media was added to the insert media at a dilution ratio of 1:50 and incubated for 24 hours. (D) qPCR analysis of human cardiac fibroblasts from (C) for the detection of transcript enrichment of *Nqo1* and *Gclc1*. The control was MRS bacterial growth media alone. Statistical significance was determined by one-way ANOVA followed by Tukey's post hoc test; the values are mean ± SEM, *n* = 5, *P < .05, **P < .01, ***P < .001.

To investigate the molecular mechanisms underlying the cardioprotective effects of LLC, we focused on Nrf2, a key transcriptional regulator of antioxidant and cytoprotective responses.^15^^,^^23^ Previous work from our group demonstrated that LLC activates Nrf2 signaling in models of intestinal injury.^14^ Extending these findings to the cardiovascular system, we first performed immunofluorescence analysis on cardiac tissue from germ-free (GF) C57BL/6 mice treated with LLC via oral gavage. Within 4 hours of treatment, we observed marked stabilization and nuclear localization of Nrf2 in cardiac tissue, indicating rapid activation of the pathway (Figure 2A). To further validate Nrf2 activation, we quantified the expression of canonical Nrf2 target genes, including *Nqo1*, *Trx1*, and *GstA1*, in cardiac tissue from LLC-treated mice. All three transcripts were significantly upregulated compared to controls, which was consistent with the transcriptional activation of the Nrf2 pathway in the heart (Figure 2B). To assess whether LLC-derived factors could influence cardiac cells indirectly via gut–epithelial signaling, we employed a transwell coculture system. Human cardiac fibroblasts were cultured beneath human intestinal epithelial monolayers and exposed to LLC-conditioned media applied to the apical surface (Figure 2C). This treatment led to increased expression of Nrf2-responsive genes in the cardiac fibroblasts (Figure 2D), suggesting that microbial metabolites, or host-derived mediators can transmit protective signals across epithelial barriers. Together, these findings demonstrate that oral LLC supplementation likely activates the Nrf2-dependent antioxidant signaling cascade in cardiac tissue, despite its anatomical separation from the gut. This suggests LLC may mediate systemic cytoprotective effects relevant to myocardial injury and repair through Nrf2 mediated mechanisms.

### Probiotic administration in the context of a Western diet driving a distinct cardiac metabolome

**Figure 3.**
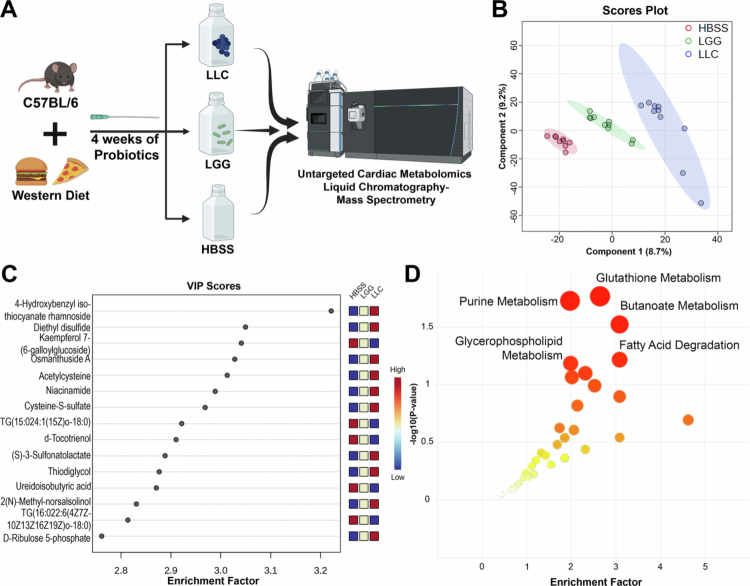
Probiotic administration in the context of a Western diet drives a distinct cardiac metabolome. Untargeted metabolomics was performed on whole-heart tissue from C57BL/6 mice fed a Western diet and cotreated for four weeks with LLC (*n* = 10), LGG (*n* = 10), or HBSS vehicle control (*n* = 9). The analysis used median-summarized unannotated high-resolution feature tables, comprising a total of 11,153 mass-to-charge (*m/z*) features. (A) Experimental design schematic illustrating diet and probiotic exposure, followed by endpoint cardiac untargeted metabolomics. (B) Partial least squares discriminant analysis (PLS-DA) based on deduplicated, xMSannotator-annotated features (3605 annotated metabolites), showing distinct separation of the HBSS (red), LGG (green), and LLC (blue) groups, reflecting treatment-specific metabolomic profiles. (C) Variable importance in projection (VIP) scores from the PLS-DA highlighting the top metabolites contributing to group separation. (D) Mummichog pathway enrichment analysis of all measured features across the three groups. Circle size represents the enrichment factor, and red intensity corresponds to statistical significance (*P*-value).

To investigate potential mechanisms by which LLC modulates cardiac metabolism and confers cardioprotection, we performed untargeted metabolomic profiling of whole-heart tissue from mice fed a Western-style diet and treated daily for four weeks with LLC (2 × 10⁹ CFU), LGG (2 × 10⁹ CFU), or vehicle control (HBSS). A Western-style diet (D12079B, Research Diets, Inc.) was selected to model dietary risk factors associated with ischemia–reperfusion (I/R) injury. At necropsy, hearts were flash-frozen, and metabolites were extracted via bead homogenization in acetonitrile containing internal standards. Extracts were analyzed using liquid chromatography–coupled high-resolution mass spectrometry (LC-HRMS) (Figure 3A). Partial least squares-discriminant analysis (PLS-DA) of putatively annotated features via xMSannotator,^24^ revealed that LLC-treated mice exhibited a distinct cardiac metabolomic profile compared to both vehicle and LGG groups, with LGG displaying an intermediate phenotype (Figure 3B). Pathway enrichment analysis using mummichog v2.0^17^^,^^25^ identified significant alterations in several metabolic pathways, including glutathione metabolism (enrichment factor [EF] = 2.203, *P*-value = 0.016), purine metabolism (EF = 1.969, *P*-value = 0.0185), butanoate metabolism (EF = 3.085, *P*-value = 0.0297), and fatty acid degradation (EF = 3.085, *P*-value = 0.0602). Notably, metabolites such as acetylcysteine, niacinamide, and cysteine-S-sulfate were enriched in LLC-supplemented hearts and contributed to group separation in the PLS-DA (Figure 3C-D; Supplementary Table S1). These findings suggest that LLC reshapes the cardiac metabolome through modulation of redox balance, nucleotide turnover, and energy metabolism, which are pathways that may underlie its observed cardioprotective effects.

### LLC supplementation modulates distinct cardiac metabolites in mice fed a Western diet

**Figure 4.**
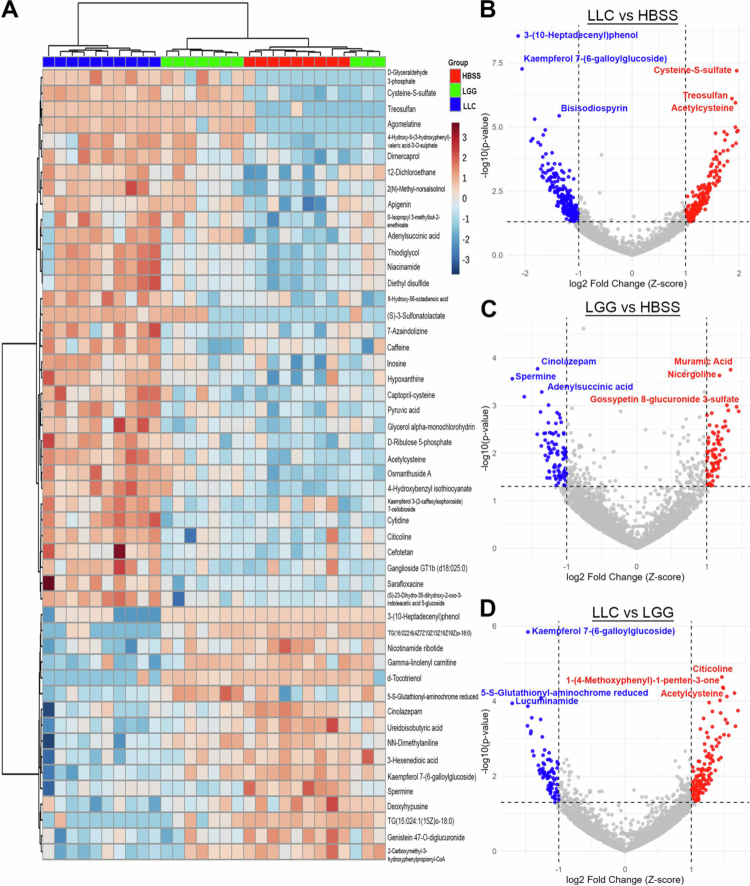
*Lactococcus lactis* subsp. *Cremoris* (LLC) supplementation modulates distinct cardiac metabolites in mice fed a Western diet. Metabolomics analysis was restricted to features annotated by xMSannotator and deduplicated by selecting the feature with the lowest ANOVA *P*-value per metabolite. (A) Heatmap of the top 50 metabolites clustered using Ward's method. *P* values for differential abundance were determined by ANOVA. (B) Volcano plot comparing LLC- versus HBSS-treated mice. Red points indicate metabolites enriched in LLC, blue points indicate metabolites depleted. Metabolites shown have |log₂ fold change| > 1 and *P* < 0.05. The top three enriched and depleted metabolites are labeled with their xMSannotator annotations. (C) Volcano plot comparing LGG- versus HBSS-treated mice, with red indicating enrichment and blue indicating depletion in LGG-treated mice. (D) Volcano plot comparing LLC- versus LGG-treated mice, with red indicating metabolites enriched in LLC relative to LGG, and blue indicating metabolites depleted in LLC relative to LGG.

To further assess how individual metabolites differed between HBSS, LGG, and LLC groups, the top 50 metabolites were analyzed by ANOVA followed by hierarchical clustering. This revealed two distinct metabolite populations: the first comprising 34 metabolites relatively enriched, and the second comprising 16 metabolites relatively depleted in LLC-treated mice compared with both LGG and control. At the group level, clustering showed LGG to cluster more closely with vehicle control (Figure 4A). Among putatively annotated metabolites enriched in LLC-treated mice, the largest groups were cysteine-containing carboxylic acid derivatives (e.g. cysteine S-sulfate, acetylcysteine) and organooxygen compounds (e.g. D-glyceraldehyde 3-phosphate, D-ribulose 5-phosphate). Several flavonoids, including apigenin and kaempferol glycosides, were also enriched, along with bioactive compounds such as caffeine, inosine, hypoxanthine, and citicoline. In addition, sulfur-containing metabolites (e.g. dimercaprol, diethyl disulfide, and thiodiglycol) were enriched in LLC-treated mice. Conversely, depleted metabolites were dominated by lipid-related classes, particularly glycerolipids (e.g. TG(16:0_22:6_18:0), TG(15:0_24:1_18:0)) and fatty acyls (e.g. *γ*-linolenyl carnitine, 3-hexenedioic acid). Depletion was also observed for prenol lipids (e.g. d-tocotrienol) and organonitrogen compounds such as spermine and *N*,*N*-dimethylaniline. Several flavonoids and isoflavonoids, including kaempferol 7-(6-galloylglucoside) and genistein diglucuronide, were also decreased in LLC-treated mice (Figure 4A-D, Table 1). Together, these data reinforce that LLC treatment, in the context of a Western-style diet, shifts metabolic programming toward alterations in energy, nucleotide, and redox metabolism, accompanied by pronounced changes in cardiac lipid metabolism.

**Table 1.** The top 50 metabolites listed in Figure 4A. Table include Putative Metabolite Annotation (Metabolite), the mass to charge ratio (m/z), the retention time, whether it is enriched or depleted in LLC treated mice, the annotation's HMDB Class, and HMDB Subclass.

#	Metabolite	m/z__retention time	Enriched/depleted in LLC	HMDB class	HMDB subclass
1	D-Glyceraldehyde 3-phosphate	175.0028__271.3	Enriched	Organooxygen compounds	Carbohydrates and carbohydrate conjugates
2	Cysteine-S-sulfate	110.0087__258	Enriched	Carboxylic acids and derivatives	Amino acids
3	Treosulfan	140.0132__280.4	Enriched	Organic sulfonic acids and derivatives	Not specified
4	Agomelatine	266.1152__84.1	Enriched	Carboxylic acids and derivatives	Not specified
5	4-Hydroxy-5-(3-hydroxyphenyl)-valeric acid-3-O-sulfate	146.0299__258.2	Enriched	Hydroxy acids and derivatives	Not Specified
6	Dimercaprol	142.035__259.7	Enriched	Thiols	Alkylthiols
7	12-Dichloroethane	181.0285__279.6	Enriched	Organochlorides	Not specified
8	2(*N*)-Methyl-norsalsolinol	180.1019__38.7	Enriched	Tetrahydroisoquinolines	Not specified
9	Apigenin	271.0595__206.9	Enriched	Flavonoids	Flavones
10	S-isopropyl 3-methylbut-2-enethioate	123.0634__36.6	Enriched	Fatty Acyls	Fatty acyl thioesters
11	Adenylsuccinic acid	486.0634__293.1	Enriched	Benzene and substituted derivatives	Biphenyls and derivatives
12	Thiodiglycol	124.0524__36.9	Enriched	Organosulfur compounds	Not specified
13	Niacinamide	123.0552__37.5	Enriched	Pyridines and derivatives	Pyridinecarboxylic acids and derivatives
14	Diethyl disulfide	123.0299__36.3	Enriched	Organic disulfides	Dialkyldisulfides
15	8-Hydroxy-56-octadienoic acid	157.0861__213.4	Enriched	Hydroxy acids and derivatives	Medium-chain hydroxy acids and derivatives
16	(S)-3-Sulfonatolactate	188.023__271.9	Enriched	Organic sulfonic acids and derivatives	Organosulfonic acids and derivatives
17	7-Azaindolizine	122.0475__36.9	Enriched	Pyrrolopyrazines	Not Specified
18	Caffeine	195.0878__291.4	Enriched	Imidazopyrimidines	Purines and purine derivatives
19	Inosine	269.0878__48.3	Enriched	Purine nucleosides	Not Specified
20	Hypoxanthine	138.0492__47.3	Enriched	Imidazopyrimidines	Purines and purine derivatives
21	Captopril-cysteine	359.0689__235.1	Enriched	Carboxylic acids and derivatives	Amino acids, peptides, and analogs
22	Pyruvic acid	171.0764__115.6	Enriched	Keto acids and derivatives	Alpha-keto acids and derivatives
23	Glycerol alpha-monochlorohydrin	111.0203__209.8	Enriched	Halohydrins	Chlorohydrins
24	D-ribulose 5-phosphate	253.0084__86.8	Enriched	Organooxygen compounds	Carbohydrates and carbohydrate conjugates
25	Acetylcysteine	186.0187__280.1	Enriched	Carboxylic acids and derivatives	Amino acids, peptides, and analogs
26	Osmanthuside A	265.1117__83.3	Enriched	Cinnamic acids and derivatives	Hydroxycinnamic acids and derivatives
27	4-Hydroxybenzyl isothiocyanate	209.9962__54.7	Enriched	Phenols	1-hydroxy-2-unsubstituted benzenoids
28	Kaempferol 3-(2-caffeoylsophoroside) 7-cellobioside	1114.3211__287.3	Enriched	Flavonoids	Flavonoid glycosides
29	Cytidine	285.1203__297.8	Enriched	Pyrimidine nucleosides	Not Specified
30	Citicoline	489.1144__294.7	Enriched	Pteridines and derivatives	Pterins and derivatives
31	Cefotetan	593.033__270.2	Enriched	Lactams	Beta lactams
32	Ganglioside GT1b (d18:025:0)	1114.6075__288.9	Enriched	Sphingolipids	Glycosphingolipids
33	Sarafloxacin	386.1317__100.9	Enriched	Phenylquinolines	Phenylquinolines
34	(S)-23-Dihydro-35-dihydroxy-2-oxo-3-indoleacetic acid 5-glucoside	430.0706__210.6	Enriched	Organooxygen compounds	Carbohydrates and carbohydrate conjugates
35	3-(10-Heptadecenyl)phenol	166.1528__292.7	Depleted	Phenols	1-hydroxy-4-unsubstituted benzenoids
36	TG(16:022:6(4Z7Z10Z13Z16Z19Z)o-18:0)	467.917__36.7	Depleted	Glycerolipids	Triradylcglycerols
37	Nicotinamide ribotide	335.064__278.4	Depleted	Pyridine nucleotides	Nicotinamide nucleotides
38	Gamma-linolenyl carnitine	422.3264__32.6	Depleted	Fatty Acyls	Fatty acid esters
39	d-Tocotrienol	397.31__34.5	Depleted	Prenol lipids	Quinone and hydroquinone lipids
40	5-S-Glutathionyl-aminochrome reduced	270.1008__107.1	Depleted	Carboxylic acids and derivatives	Amino acids, peptides, and analogs
41	Cinolazepam	737.1268__256	Depleted	Benzodiazepines	1,4-benzodiazepines
42	Ureidoisobutyric acid	147.5549__89.7	Depleted	Organic carbonic acids and derivatives	Ureas
43	NN-Dimethylaniline	122.0964__39.3	Depleted	Organonitrogen compounds	Amines
44	3-Hexenedioic acid	167.0316__55.7	Depleted	Fatty Acyls	Fatty acids and conjugates
45	Kaempferol 7-(6-galloylglucoside)	664.1288__290.4	Depleted	Flavonoids	Flavans
46	Spermine	203.223__20.4	Depleted	Organonitrogen compounds	Amines
47	Deoxyhypusine	218.1864__198.8	Depleted	Carboxylic acids and derivatives	Amino acids, peptides, and analogs
48	TG(15:024:1(15Z)o-18:0)	467.9637__47.3	Depleted	Glycerolipids	Triradylcglycerols
49	Genistein 47-O-diglucuronide	664.1495__256.6	Depleted	Isoflavonoids	Isoflavonoid O-glycosides
50	2-Carboxymethyl-3-hydroxyphenylpropionyl-CoA	1018.1439__257.3	Depleted	Fatty Acyls	Fatty acyl thioesters

**Figure 5.**
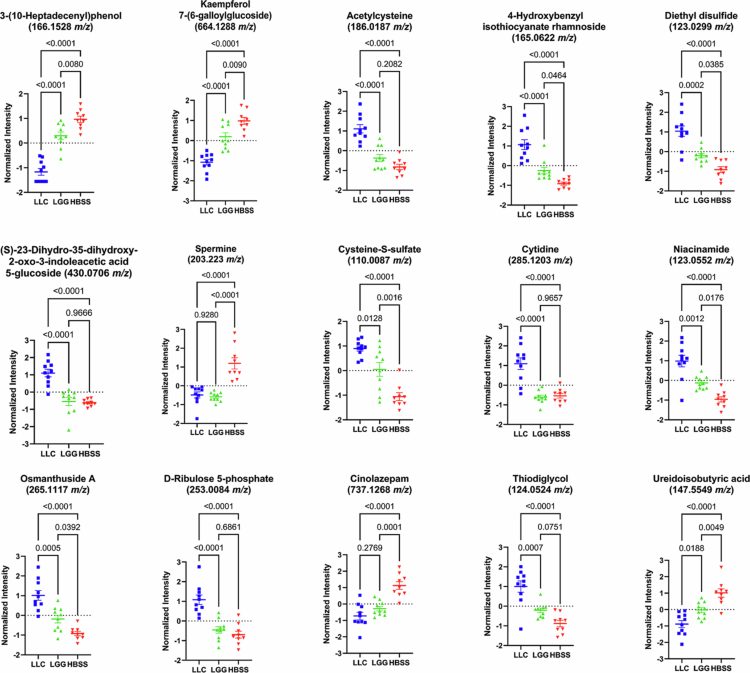
The top 15 differentially altered cardiac metabolites identified by ANOVA. Metabolomics analysis was restricted to features annotated by xMSannotator and deduplicated. Putative metabolite annotations are shown, with corresponding mass-to-charge ratios (*m/z*) indicated below each annotation. Color coding represents treatment groups: blue = LLC, green = LGG, red = vehicle.

Analysis of the top differentially abundant metabolites revealed clear group-dependent patterns, with LLC treatment driving a distinct metabolic profile compared to LGG and HBSS controls (Figure 5). Several metabolites, including 3-(10-Heptadecenyl)phenol, kaempferol 7-(6-galloylglucoside), spermine, and uredoisobutyric acid were markedly depleted in LLC relative to both LGG and HBSS. In contrast, LLC mice exhibited pronounced enrichment of sulfur-containing metabolites such as acetylcysteine, diethyl disulfide, cysteine-S-sulfate, and thiodiglycol, as well as nucleotide-related metabolites including cytidine, niacinamide, and D-ribulose 5-phosphate. Interestingly LGG treated mice consistently demonstrated intermediate metabolite intensity measures when compared to LLC and control. LLC treated mice consistently diverged, characterized by broad suppression of plant-derived polyphenols and amino acid derivatives alongside enrichment in thiol and nucleotide metabolism related features (Figure 5). Together, these findings indicate that LLC elicits a more pronounced reprogramming of host metabolism than LGG, favoring pathways linked to sulfur turnover, redox regulation, and nucleotide biosynthesis.

### Weighted correlation network analysis (WGCNA) reveals distinct cardiac metabolite modules affected by probiotic supplementation

**Figure 6.**
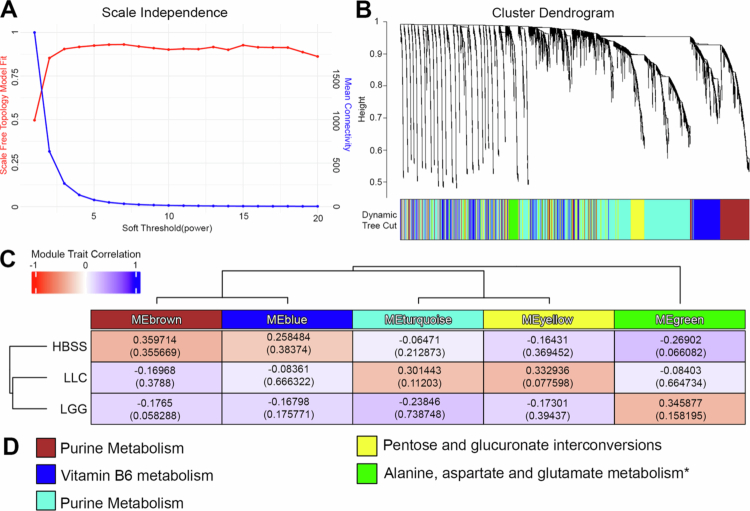
Weighted correlation network analysis (WGCNA) revealed distinct cardiac metabolite modules affected by probiotic supplementation. WGCNA was performed on all detected features (annotated and unannotated) using a minimum module size of 250 features, a soft-thresholding power of 4, and a deep split of 2. (A) Scale-free topology model fit (R², red line) and mean connectivity (blue line) across candidate soft-thresholding powers, used to select the optimal soft power. (B) Hierarchical clustering dendrogram of metabolites with module color assignments. Modules were identified using the dynamic tree cut method applied to an average linkage hierarchical clustering tree based on the topological overlap matrix (TOM). Metabolites not assigned to any module (gray) were excluded, resulting in five modules. (C) Hierarchical clustering of modules (top) and module–trait correlation heatmap (bottom). Each row corresponds to a probiotic treatment, and each column represents a module eigengene summarizing the abundance pattern of metabolites within that module. (D) Metabolic pathway with the highest enrichment factor identified by mummichog pathway enrichment analysis for features within each module. The analysis was restricted between highest correlation value and either LLC or HBSS as appropriate. *Indicates all three experimental groups were utilized in mummichog software due to failure of pathway analysis using pairwise comparisons.

To elucidate coregulated metabolite modules and characterize system-level perturbations following probiotic administration, we performed WGCNA on the unannotated raw feature set. To establish the appropriate network construction parameters, we first evaluated the scale-free topology fit and mean connectivity across candidate soft-thresholding powers, identifying a power of 4 as optimal for downstream analysis (Figure 6A). Using this threshold and a minimum cluster size of 250, hierarchical clustering of all detected metabolic features followed by dynamic tree cutting revealed five distinct metabolite modules, with unassigned features grouped into the gray module (Figure 6B). Module‒trait correlation analysis revealed treatment-specific associations between module eigengenes and probiotic treatments (Figure 6C). The features left within each module were then used in mummichog v2.0 software for pathway analysis, limiting analysis between the highest correlated treatment group and either HBSS or LLC when appropriate. The MEbrown eigengene was positively associated with HBSS, reflecting higher abundances of metabolites in this module under HBSS conditions. Pathway enrichment between HBSS and LLC revealing changes most notable in purine metabolism (EF = 8.213, *P* value = 0.116). Similarly, Meblue was associated with HBSS and pathway enrichment between HBSS and LLC revealing changes most notable in vitamin B6 metabolism (EF = 20.749, *P* value = 0.048). MEturquoise and MEyellow were positively associated with LLC, with pathway enrichment between LLC and HBSS revealing changes most notable in purine metabolism and pentose and glucuronate interconversions, respectively. Finally, MEgreen was associated with LGG treatment, and pathway enrichment was most notable for alanine, aspartate and glutamate metabolism (EF = 13.00, *P* value = 0.074) (Figure 6D, Supplementary Table S2).

### Probiotic administration results in distinct changes in gut-associated metabolites

**Figure 7.**
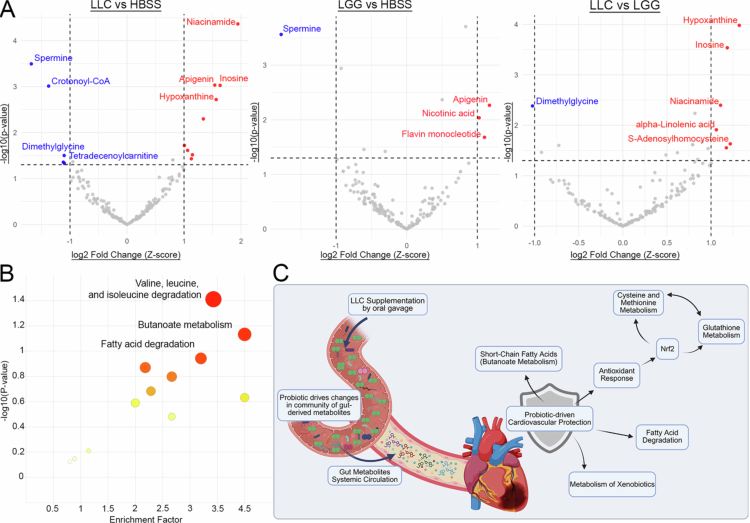
Probiotic administration results in distinct changes in gut-associated metabolites. Cross-referencing annotations from xMSannotator with the Microbial Metabolites Database (MiMeDB) identified 171 annotated features from the original 11,153 measured mass-to-charge (*m/z*) ratios. (A) Volcano plots showing significantly altered metabolites between three comparisons: LLC vs. HBSS, LGG vs. HBSS, and LLC vs. LGG. Significance thresholds were defined as |log₂ fold change| > 1 and *P* < .05. (B) Mummichog pathway enrichment analysis for metabolites differing between LLC and HBSS. Other comparison groups did not yield sufficient significantly altered metabolites for pathway analysis. (C) Graphical abstract depicting potential mechanisms of cardiovascular protection mediated by LLC administration.

To gain a better perspective on how LLC is shaping the gut-associated metabolites in the heart, our annotated metabolite feature set consisting of 3605 putatively annotated metabolites by xMSannotator was cross-referenced those with a 1162 gut-associated metabolite reference database curated from The Human Microbial Metabolome Database (MiMeDB).^22^ This analysis identified a gut-metabolite feature set of 171 metabolites. Pairwise comparisons across the three experimental groups revealed that LLC treatment induced the most pronounced metabolic changes, with significant enrichment of Niacinamide (log2FC = 1.94, *P*-value = 4.33 × 10⁻⁵), apigenin, inosine, hypoxanthine, 1,3,7-trimethyluric acid, tartaric acid, dehydro-*p*-cymene, and S-adenosylhomocysteine, alongside depletion of spermine, crotonoyl-CoA, dimethylglycine, tetradecenoylcarnitine, and FAD relative to HBSS. Comparisons between LLC and LGG revealed a similar pattern, with enrichment of niacinamide, hypoxanthine, inosine, alpha-linolenic acid, PC (20:4/18:1), and S-adenosylhomocysteine and depletion of dimethylglycine and tetradecenoylcarnitine, suggesting modulation of nucleotide, lipid, and methylation pathways. Compared with HBSS, LGG treatment elicited more modest changes, with enrichment of apigenin, nicotinic acid, and flavin mononucleotide and depletion of spermine, indicating subtler metabolic effects. Notably, niacinamide was consistently elevated in LLC-treated samples but unchanged with LGG alone, highlighting an LLC-specific impact on NAD⁺ metabolism, whereas spermine depletion occurred with both probiotic treatments (Figure 7A). Pathway enrichment analysis revealed that, for the LLC vs HBSS comparison, several relevant metabolic pathways were significantly enriched, including valine, leucine, and isoleucine degradation (EF = 4.987, *P* value = 0.03), butanoate metabolism (EF = 3.74, *P* value = 0.03), and fatty acid degradation (EF = 4.363, *P* value = 0.062) (Figure 7B, Supplementary Table S3). No significant pathway enrichment was observed in the other pairwise comparisons. Collectively, these findings underscore the salient role of LLC as a probiotic intervention capable of modulating systemic gut-derived metabolites, with potential downstream effects on antioxidant capacity, xenobiotic metabolism, and fatty acid degradation (Figure 7C).

## Discussion

Our findings reveal that LLC exerts significant cardioprotective effects in preclinical models of myocardial injury, expanding its known therapeutic profile beyond the gut and the liver. The observed improvements in cardiac function and reductions in myocardial scarring following LLC supplementation suggest a systemic mechanism of action, potentially mediated through gut‒heart axis signaling. Metabolomic profiling of cardiac tissue uncovered distinct shifts in metabolic pathways, including enhanced glutathione metabolism and fatty acid degradation, both of which are critical for maintaining redox balance and energy homeostasis in the injured myocardium.^26-29^ These data support a model in which LLC promotes metabolic reprogramming and anti-inflammatory signaling, thereby mitigating maladaptive remodeling and preserving cardiac function postinfarction.^30^

Beyond its structural and functional benefits to the myocardium, LLC supplementation induced profound shifts in the cardiac metabolome, suggesting a systemic reprogramming of host metabolic pathways. Untargeted metabolomic profiling revealed enrichment of glutathione metabolism, a key antioxidant pathway that protects cardiomyocytes from oxidative stress during ischemia–reperfusion injury.^27^^,^^29^ Enhanced fatty acid degradation further points to preserved mitochondrial efficiency and energy utilization, which are critical for maintaining cardiac output under stress conditions.^28^ In addition, the hearts of LLC-treated animals exhibited alterations in amino acid and lipid biosynthesis pathways, indicating broader metabolic remodeling that may support tissue repair and limit fibrotic remodeling.^31-33^ These changes are consistent with LLC's known activation of the Nrf2 pathway in the gut, suggesting that its systemic effects may be mediated through conserved cytoprotective signaling cascades. Network-based metabolomics further revealed that these effects occur at the level of coordinated metabolite modules rather than isolated features. Specifically, modules positively associated with LLC were enriched for purine metabolism and pentose/glucuronate interconversions, reflecting increased nucleotide turnover and carbohydrate flux to support energy production and redox balance under cardiac stress.^20^^,^^34-36^ In contrast, modules associated with HBSS were enriched for vitamin B6 metabolism, a cofactor pathway largely reflective of basal nutrient turnover and housekeeping function, which may imply a more unperturbed metabolic state.^37^ Meanwhile, the LGG-associated module was enriched for alanine, aspartate, and glutamate metabolism, suggesting intermediate remodeling centered on amino acid catabolism, which may provide modest anaplerotic support to the TCA cycle but does not achieve the broader reprogramming observed with LLC.^33^^,^^36^^,^^38^ Taken together, these findings underscore that LLC supplementation drives coordinated, systems-level rewiring of cardiac metabolism, engaging pathways that align with cytoprotective and reparative processes, whereas HBSS and LGG maintain metabolic states more reflective of baseline or partial adaptation. Importantly, the enrichment of nucleotide- and redox-associated modules in LLC-treated hearts provides a mechanistic link to the observed reductions in scar formation and preservation of systolic function, suggesting that metabolic network reprogramming directly underpins the structural and functional cardioprotection conferred by LLC.

Our cross-referencing of the cardiac metabolome against a database of gut-associated metabolites (MiMeDB)^22^ revealed that a distinct subset of microbiota-associated metabolites was altered by LLC supplementation. This provides salient evidence that probiotic administration can shape the systemic distribution of microbial metabolites, with functional consequences in distal organs such as the heart. Several of the enriched metabolites have well-described roles in cardiovascular biology: niacinamide supports NAD⁺ metabolism and mitochondrial redox capacity,^39-42^ inosine and hypoxanthine contribute to purine salvage and immunomodulation,^43^ and S-adenosylhomocysteine reflects altered methylation flux, with implications for epigenetic and redox regulation.^44^ Conversely, depletion of metabolites such as spermine and acyl-carnitines in LLC-treated mice suggests remodeling of polyamine^45^ and lipid metabolism, both of which are linked to maladaptive remodeling in the injured myocardium.^46^^,^^47^ Notably, niacinamide was selectively elevated in LLC, but not LGG-treated animals, underscoring that probiotic effects on the gut–heart axis are species-specific, since LGG was used primarily to control for effects of lactic acid fermentation potential.^48^ Importantly, we conducted targeted MS/MS analyzes using authentic standards for niacinamide, nicotinic acid, and spermine independently on an IDX mass spectrometry system. Retention times for each metabolite were confirmed within 10 seconds of the standards, and MS¹/MS² fragmentation spectra showed excellent concordance (Supplementary Figure 2). Together, these findings support a model in which LLC promotes cardioprotection not only through host-intrinsic pathways (e.g. Nrf2 activation), but also through reshaping of the systemic gut-derived metabolite pool, providing circulating metabolites that enhance mitochondrial efficiency, redox resilience, and epigenetic regulation in the myocardium.

This study provides new insights into the cardioprotective effects of LLC, highlighting its ability to modulate host metabolism and activate protective signaling pathways, including Nrf2. While the data infers a link between LLC treatment and improved cardiac outcomes, the evidence remains correlative. Future studies employing targeted supplementation or depletion of candidate metabolites will be essential to determine whether these molecules act as active mediators or serve as biomarkers of LLC activity. Similarly, while Nrf2 activation emerged as a key early response to LLC, the current study lacks direct causal evidence. This is challenging because an approach such as pharmacological inhibition of Nrf2 is confounded by off-target effects, and systemic Nrf2 knockout models introduce global oxidative stress that complicates interpretation. Given these limitations, we relied primarily on associative data and propose that Nrf2 activation may serve as a priming event that initiates downstream protective cascades, with full cardioprotection requiring sustained engagement of these pathways. Further studies employing cardiomyocyte-specific Nrf2 knockout mice would help validate the mechanistic requirement of Nrf2 for LLC-mediated cardiac cytoprotection.^49^ To dissect the immediate versus long-term effects of LLC, we employed both GF and conventionally raised mice. GF mice allowed for the isolation of the direct impact of LLC in the absence of a resident microbiota, revealing rapid Nrf2 activation within 4 hours of gavage. This supports the hypothesis that LLC-derived metabolites or surface molecules can directly engage host signaling pathways. Conventionally raised mice were used for longer-term I/R injury studies to assess LLC's efficacy in a physiologically relevant microbial context. While effects were more pronounced in GF mice, beneficial trends in conventionally raised mice show retained activity in microbial colonized mice.

The inclusion of LGG as a comparator strain facilitated characterization of the specificity of LLC's effects. Although LGG shares general probiotic properties, it failed to replicate LLC's impact on the metabolome, underscoring the importance of strain-specific metabolic capabilities and host interactions. From a translational perspective, our findings suggest that LLC holds promise as a microbiome-based intervention for ischemic heart disease. Advancing LLC toward clinical application will require addressing key considerations, including safety, dosing, delivery, and efficacy across diverse contexts and populations. Personalized approaches that account for microbiome composition and sex-specific responses will be critical. Compared to other probiotic strains previously tested in heart failure models, including LGG,^50^ *Bifidobacterium longum*,^51^^,^^52^ and *Akkermansia muciniphila*,^53^ LLC also demonstrated a robust preservation of cardiac function and distinct molecular signatures. Of further note, butyrate-producing bacteria have also demonstrated postinfarction benefits in preclinical models, reinforcing the concept that multiple microbial metabolites may contribute to cardioprotection.^54^

The limitations of the current study are as follows. First, we note that the EF and FS values observed in our I/R model at 1 week following injury are somewhat milder than those typically reported in the literature.^55^ This difference may be attributed to multiple factors, including variability in surgical precision during the 30-minute LAD ligation and reperfusion procedure, sex differences as in our experiment we utilized young adult female C57BL/6J mice (8–12 weeks), and an optimized postoperative care regimen that minimized mortality and variability in infarct size. Nevertheless, we did detect EF and FS values comparable to those reported in the literature by four weeks following I/R in HBSS-supplemented mice, demonstrating the severity of the cardiac injury. Furthermore, in our approach, all experiments were conducted using female mice, based on prior findings demonstrating that LLC confers protection against Western diet-induced hepatic lipid accumulation and inflammation specifically in females.^16^ This choice ensured biological consistency across studies and facilitated interpretation of LLC's cardiometabolic effects. However, it is recognized that sex is a critical determinant of cardiovascular physiology and pathophysiology, and that inclusion of both sexes would enhance the generalizability of our findings. For future studies, it will be essential to evaluate LLC's efficacy in male mice and to explore potential sex-specific mechanisms. Finally, dietary composition is another important variable that may influence our findings. While I/R experiments were performed under standard chow conditions, metabolomic profiling included mice fed either standard chow or a Western-style diet. The Western style diet was used to model metabolic stress and reflect dietary patterns commonly associated with increased cardiovascular risk. We acknowledge that this introduces an additional variable that could contribute to the observed metabolomic differences independent of LLC treatment and may limit the conclusions we might draw from the effects of LLC supplementation. Nonetheless, characterizing LLC's effects under Western diet conditions provides clinically relevant insights into its therapeutic potential in metabolically challenged settings. Future studies should aim to systematically control for dietary variables to isolate treatment-specific effects. We additionally acknowledge that while we did verify the identity of several key metabolites using authenticated standards the majority of annotations in our untargeted analysis remain putative. Nevertheless, most features discussed exhibit strong computational predictive confidence (Supplementary Table S4).^24^

## Materials and methods

### Animals

All animal procedures were approved by the Emory University Institutional Animal Care and Use Committee and conducted in accordance with institutional guidelines. Conventional female C57BL/6 mice (6–8 weeks old) were obtained from Jackson Laboratories (Bar Harbor, ME) and housed under specific pathogen-free conditions with the support of the Emory Gnotobiotic Animal Core (EGAC). Germ-free mice were obtained from EGAC. Mice were provided ad libitum access to sterilized 2019 Teklad Global 19% Protein Extruded Rodent Diet (Inotiv, Indianapolis, IN), or a Western diet (D12079B, Research Diets, Inc.) as noted, and autoclaved drinking water. For select experiments involving probiotic administration to germ-free (GF) mice, mice were housed in hermetically sealed ISOcage *P*-Bioexclusion units (Tecniplast, West Chester, PA) within EGAC to maintain microbiological containment. All animals were maintained under standard environmental conditions, including a 12-hour light/dark cycle, and monitored regularly for health and behavior. Where outlined, experimental groups received daily oral gavage of probiotics or vehicle control as specified in the study design. To evaluate the impact of LLC on the cardiac health, mice were randomly assigned to receive daily oral gavage of 2 × 10^9^ CFU LLC or vehicle control (HBSS) for 2 weeks before induction of Ischemic Reperfusion (I/R).

### Ischemic reperfusion

Ischemic Reperfusion (I/R) was surgically induced in conventional adult female mice (>8 weeks old) fed a standard control chow. Anesthesia was administered via inhalation of 1%–3% isoflurane for both induction and maintenance, with depth confirmed by respiratory rate and absence of response to toe-pinch. Aseptic technique was strictly followed, with personnel wearing sterile gloves, face masks, and performing a full surgical scrub. The surgical site was shaved and cleaned with 70% ethanol. Endotracheal intubation was performed using a PE180 tube, and mice were mechanically ventilated (0.3–0.4 cc at 110–120 rpm, 1–2 L/min oxygen). A thoracotomy was initiated with lidocaine applied to the incision site, followed by a 2-cm incision on the left chest and blunt dissection through the third intercostal space to expose the heart via retraction and pericardial opening. MI was induced by ligating the left anterior descending coronary artery with an 8-0 prolene suture. Closure involved positive pressure ventilation, rib approximation with 2-0 nylon, muscle closure with 4-0 absorbable sutures, and skin closure using surgical clips. Postoperative recovery included placement under a heating lamp and monitoring every 15 minutes until ambulatory, then hourly. Analgesia was provided with Flunixin (2.5 mg/kg, 0.5 mL, subcutaneously) daily for 3 d. Health monitoring was conducted twice daily for 72 hours and daily thereafter. Before I/R surgery, and then at 1 week and 4 weeks after surgery, parasternal long-axis (PSLAX) echocardiography was performed to assess cardiac structure and function in the mice. The animals were anesthetized with 1%–2% isoflurane in oxygen to minimize movement while maintaining stable heart rates and placed supine on a heated platform to preserve body temperature. Imaging was conducted using a high-frequency ultrasound system (Vevo 3100), with the transducer positioned at a 45° angle on the left side of the sternum to obtain the PSLAX view, enabling visualization of the left ventricle, left atrium, aortic root, and mitral valve. Imaging measured left ventricular wall thickness, chamber dimensions, and fractional shortening, while Doppler imaging was employed to assess blood flow across the mitral and aortic valves when applicable. Echocardiographic data acquisition and analysis were performed by investigators blinded to treatment groups. Data were analyzed offline using vendor-provided or open-source software.

### Measuring transcript enrichment by quantitative PCR

Heart tissues from germ-free C57BL/6 mice treated or untreated with LLC were mechanically homogenized in TRIzol reagent (Invitrogen, Carlsbad, CA) using a MagnaLyser instrument equipped with MagnaLyser beads (Roche, Basel, Switzerland). Total RNA was isolated using the Aurum Total RNA Mini Kit (Bio-Rad, Hercules, CA) to ensure optimal yield and purity. Complementary DNA (cDNA) was synthesized from 1 µg of total RNA using the iScript cDNA Synthesis Kit (Bio-Rad, Hercules, CA), following the manufacturer's instructions. Quantitative real-time PCR (qPCR) was performed using the iQ SYBR Green Supermix (Bio-Rad, Hercules, CA) on a Bio-Rad CFX96 Real-Time PCR Detection System. Gene-specific primers were used to assess transcript levels, and relative expression was calculated using the ΔΔCt method, normalized to actin housekeeping gene. Primer pairs for studies in mice include *Gst1a*-F, 5′- GGGTGGAGTTTGAAGAGAAGT-3′, *Gst1a*-R, 5′- TGGCGATGTAGTTGAGAATGG-3′, *Trx1*-F, 5′-CGTGGTGGACTTCTCTGCTACGTGGTG-3′, *Trx1*-R, 5′-GGTCGGCATGCATTTGACTTCACAGTC-3′, *Nqo1*-F, 5′-GCCGAACACAAGAAGCTGGAAG-3′, *Nqo1*-R, 5′-GGCAAATCCTGCTACGAGCACT-3′.

*β*-Actin-F, 5′-AATGTGGCTGAGGACTTTGT-3′, *β*-Actin, 5′-GGGACTTCCTGTAACCACTTATT -3′. Primer pairs for studies in human cells include, *Nqo1*-F, 5′-CCTGCCATTCTGAAAGGCTGGT-3′, *Nqo1*-R, 5′-GTGGTGATGGAAAGCACTGCCT-3′, *Gclc1*-F, 5′-GGAAGTGGATGTGGACACCAGA-3′, *Gclc1*-R, 5′-GCTTGTAGTCAGGATGGTTTGCG-3′, *β*-Actin-F, 5′-CACCATTGGCAATGAGCGGTTC-3′, and *β*-Actin, 5′-AGGTCTTTGCGGATGTCCACGT-3′. All reactions were run in technical duplicates or triplicates, and melt curve analysis was conducted to confirm amplification specificity.

### Histologic analysis of mouse cardiac tissue

Mouse hearts were harvested, rinsed in phosphate-buffered saline (PBS), and fixed in 10% neutral-buffered formalin for 24 hours at room temperature. Fixed tissues were embedded in paraffin, sectioned at 5 µm thickness, and mounted on glass slides. For general histological assessment, sections were stained with Sirius Red to evaluate tissue collagen deposition and fibrosis. Furthermore, to assess myocardial fibrosis and scar formation, adjacent sections were stained using Masson's trichrome protocol, which differentiates collagen (blue), muscle fibers (red), and nuclei (black). Stained slides were imaged using a brightfield microscope (Leica DM5000 B), and quantitative analysis of fibrotic area was performed using ImageJ software. Fibrosis was expressed as the percentage of collagen-stained area relative to total myocardial area in each section.

### Western diet and probiotic exposure model

To evaluate the impact of LLC on the cardiac metabolome under dietary stress, conventional C57BL/6 mice were randomly assigned to receive daily oral gavage of LLC, LGG, or vehicle control (HBSS) while concurrently being fed a Western-style diet (D12079B, Research Diets, Inc.). This diet provides 17% of total caloric intake from protein, 43% from carbohydrates, and 40% from fat, mimicking the macronutrient composition associated with increased cardiovascular risk. Probiotic and dietary interventions were administered simultaneously for a duration of four weeks. At the end of the treatment period, mice were euthanized, and whole hearts were harvested, flash-frozen in liquid nitrogen, and stored at –80 °C for subsequent metabolomic analysis.

### High-resolution metabolomics (HRM) sample preparation

Whole heart cardiac samples from the Western diet and probiotic exposure model were prepared by the addition of 15 µl of ice-cold 33% LCMS Grade Water (Thermo Scientific 047146-K2)/66% acetonitrile-internal standard solution per milligram of sample followed by homogenization using chilled MagNA Lyser Green Beads (Roche REF 03358941001) at maximum speed (7000) for 30 seconds. The homogenized samples were transferred to microcentrifuge tubes, vortexed, and placed on ice for 30 minutes before centrifugation at 14,000 × *g* for 10 minutes at 4 °C to precipitate the proteins. The supernatants were transferred to autosampler vials and stored at −80 °C before instrumental analysis.

### HRM instrument analysis

Samples were analyzed on a dual column and pump LC system (Thermo Ultimate3000 uHPLC) connected to a high-resolution mass spectrometer (Thermo Scientific Q Exactive HF). The two analytical platforms consisted of a Waters Xbridge BEH Amide XP HILIC column (2.1 mm × 50 mm, 2.6 μm particle size) coupled with positive electrospray ionization (ESI + ) and a Higgins Targa C18 column (2.1 mm × 50 mm, 3 μm) coupled with negative electrospray ionization (ESI-). The mobile phases included LCMS-grade water (A), LCMS-grade acetonitrile (B), 2% formic acid in LCMS-grade water (C), and 10 mM ammonium acetate in LCMS-grade water (D). For the HILIC chromatography gradient, initial buffer ratios of 22.5% A, 75% B, and 2.5% C were held for 1.5 minutes before ascending to 75% A, 22.5% B, and 2.5% C for 4 minutes and ending with a 1-minute gradient hold. For the C18 chromatography gradient, an initial buffer ratio of 60% A, 35% B, and 5% C was held for 1 minute before ascending to 0% A, 95% B, and 5% C for 3 minutes and ending with a 2-minute gradient hold. Column compartments were heated to 60 °C. The flow rates were set to 0.35 mL/min for the first minute and 0.4 mL/min for the remaining 4 minutes. Mass spectra were collected at 120k resolution in an 85–1275 *m/z* scan window.

### HRM data processing and statistics

Peak detection, noise removal, alignment and quantification were performed using adaptive processing for LCMS data (apLCMS) v6.3.3,^56^ with downstream quality control performed by xMSanalyzer v.2.0.8.^24^ Each metabolic feature was characterized by its *m/z* ratio, retention time, and peak intensity. For pathway enrichment analysis, differentially expressed features were normalized by log transformation, mean-centered and divided by the square root of the standard deviation of each variable and the top 10% of peaks used in mummichog v2.0 software.^25^ Spearman correlations and heatmaps were performed and prepared using Metaboanalyst v6.0.^31^^,^^57^ For volcano plots, metabolomics data were imported and reshaped using the tidyverse suite in R. Intensities were normalized to z-scores within each metabolite feature. Pairwise group comparisons were performed by calculating fold changes of mean z-scores and conducting two-sided t-tests for differential abundance. Features with log2 fold change > 1 and *p* < 0.05 were considered significant. Volcano plots highlighting enriched and depleted metabolites were generated using ggplot2.

### HRM metabolite annotation

Detected features were annotated using xMSannotator.^24^ All annotated features were used for downstream analysis, with the exception of the mummichog conducted in Figure 3D and WGCNA analysis in Figure 6, where all measured features were used in pathway enrichment analysis. Duplicate annotations were dealt with by excluding the annotations with higher *P* values via ANOVA. Annotations were further restricted via comparison against an in-house reference library established with authentic chemical standards and matched within 5 ppm of the confirmed mass and within tens of the confirmed retention times. Furthermore, annotations were restricted via cross-reference with the Microbial Metabolites Database (MiMeDB)^22^ for analysis conducted in Figure 7.

### Weighted gene coexpression network analysis (WGCNA)

Median-summarized, m/z-calibrated untargeted metabolomics data were processed in R (v4.3.3) using the WGCNA package (v1.72-5).^19^ Features were labeled by m/z and retention time (mz__rt), log-transformed, and filtered using *goodSamplesGenes* to remove low-quality samples and features. Hierarchical clustering was used to assess sample relationships, and the soft-thresholding power (*β* = 4) was chosen using *pickSoftThreshold* to approximate scale-free topology. Pearson correlations were converted to a topological overlap matrix (TOM), and metabolites were clustered by average linkage on 1-TOM. Modules were detected using dynamic tree cutting (deepSplit = 2) at minClusterSize values of 100, 250, and 500 to assess granularity. A cluster size of 250 was ultimately used for downstream analysis. Module eigengenes were correlated with experimental traits (treatment groups) to identify biologically relevant modules, with significance determined by Student's t test. Module assignments, eigengenes, correlations, and annotated feature lists were exported for downstream interpretation.

### Statistics

Statistical analysis was performed using Prism 10 (GraphPad, San Diego, CA). Significance is defined as *P ≤ .05, **P ≤ .01, ***P ≤ .001, ****P ≤ .0001.

## Supplementary Material

Gacasan_et_al_Supplementary_Data_12_19_2025.docx Gacasan_et_al_Supplementary_Data_12_19_2025.docx

## Data Availability

Raw data not included in this article are available on Metabolomics Workbench. Metabolomics Workbench DOIs for these studies are pending. The median summarized feature tables and project metadata can be found: Gacasan, C.A. (2025) “gacasan_LLCcardioprotection_2025”. Zenodo. doi:10.5281/zenodo.16921447.
