# Supplementary material for: Lactococcus lactis subsp. Cremoris reprograms systemic metabolism and protects against myocardial injury
Source: Gut Microbes. 2026 Jan 4;18(1):2609426. doi: 10.1080/19490976.2025.2609426 (PMC12773551; doi:10.1080/19490976.2025.2609426)
Supplement: Gacasan_et_al_Supplementary_Data_12_19_2025.docx [file KGMI_A_2609426_SM0716.docx]

***Lactococcus lactis* subsp. Cremoris Reprograms Systemic Metabolism and Protects Against Myocardial Injury**

**SUPPLEMENTARY DATA**

C. Anthony Gacasan^1^, Crystal R. Naudin^1^, Jaclyn Weinberg^2^, Lauren C. Askew^1^, Maria E Barbian^3^, Dean P. Jones^2^, and Rheinallt M. Jones^1^*

^1^Division of Gastroenterology, Hepatology, and Nutrition, ^2^Division of Pulmonary, Allergy, Critical Care and Sleep Medicine, Department of Medicine, Department of Pediatrics, ^3^Division of Neonatology, Department of Pediatrics, Emory University School of Medicine, Atlanta GA, 30322.

**Corresponding author:** ^*^ Rheinallt M. Jones, Division of Gastroenterology, Hepatology, and Nutrition, Department of Pediatrics, Emory University School of Medicine, 615 Michael Street, Atlanta GA, 30322, (rjones5@emory.edu) Tel: (404) 727-7231, Fax: (404) 727-8538

***Key words:*** Gut-heart axis, *Lactococcus lactis* subsp. Cremoris, Probiotic, atherosclerosis, myocardial infarction, Metabolomics,

**Running head:** LLC Reprograms Metabolism and Protects Against Myocardial Injury

**Supported** by the National Institutes of Health grants F30DK139762 (CAG), F30DK134204 (LCA), and K12HD072245 (MEB).


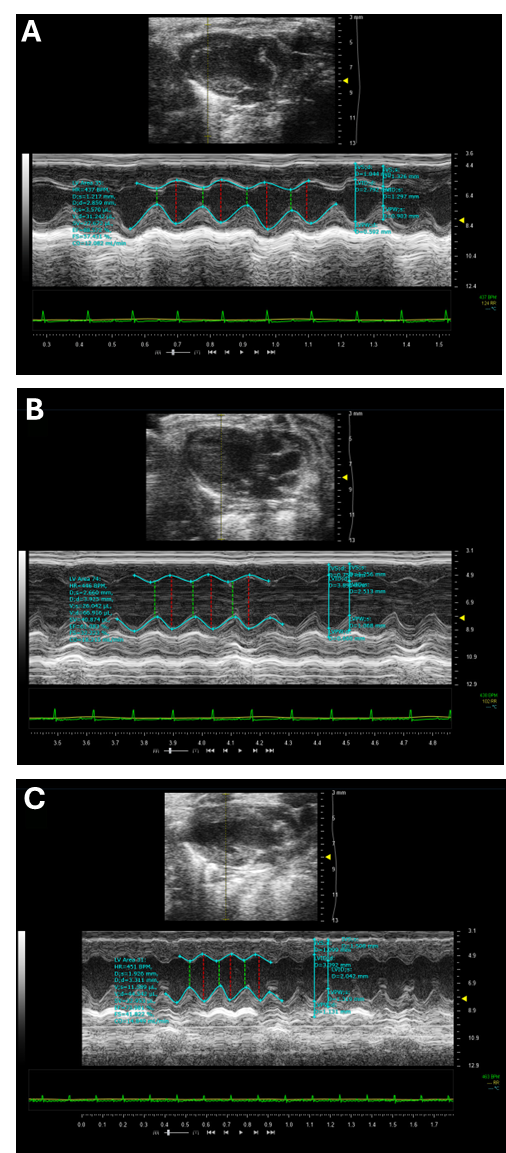
**Supplementary Figure 1.** Representative Parasternal Long Axis (PSLAx) echocardiographic views used to assess cardiac chambers, valves, and overall function in mice subjected to the ischemia/reperfusion (I/R) procedure. **(A)** PSLAx view of a sham-operated mouse prior to I/R. **(B)** PSLAx view of an HBSS-supplemented mouse at 4 weeks post-I/R. **(C)** PSLAx view of an LLC-supplemented mouse at 4 weeks post-I/R.

| **Median Summarized Pathway Enrichment Analysis (LLCvsLGGvsHBSS)** | | | | | | |
| --- | --- | --- | --- | --- | --- | --- |
|  | **Pathway total** | **Hits.total** | **Enrichment Factor** | **Hits.sig** | **Expected** | **P(Fisher)** |
| **Glutathione metabolism** | 19 | 11 | 2.20342 | 5 | 2.2692 | 0.016928 |
| **Purine metabolism** | 70 | 32 | 1.969047 | 10 | 5.0786 | 0.018547 |
| **Butanoate metabolism** | 15 | 10 | 3.084754 | 4 | 1.2967 | 0.029724 |
| **Fatty acid degradation** | 35 | 8 | 3.084833 | 3 | 0.9725 | 0.06025 |
| **Glycerophospholipid metabolism** | 13 | 7 | 3.084833 | 3 | 0.9725 | 0.06025 |
| **Cysteine and methionine metabolism** | 33 | 20 | 1.652619 | 5 | 3.0255 | 0.064911 |
| **Valine, leucine and isoleucine degradation** | 35 | 14 | 2.892012 | 5 | 1.7289 | 0.079077 |
| **Alanine, aspartate and glutamate metabolism** | 28 | 15 | 1.609464 | 4 | 2.4853 | 0.085566 |
| **Primary bile acid biosynthesis** | 46 | 11 | 1.682652 | 2 | 1.1886 | 0.10154 |
| **Caffeine metabolism** | 10 | 5 | 4.627273 | 3 | 0.64833 | 0.12611 |
| **Metabolism of xenobiotics by cytochrome P450** | 68 | 13 | 2.847583 | 4 | 1.4047 | 0.15124 |
| **Terpenoid backbone biosynthesis** | 14 | 2 | 4.627273 | 1 | 0.21611 | 0.20163 |
| **Pentose phosphate pathway** | 23 | 15 | 4.62722 | 8 | 1.7289 | 0.23667 |
| **Pentose and glucuronate interconversions** | 18 | 6 | 3.084833 | 3 | 0.9725 | 0.24676 |
| **Inositol phosphate metabolism** | 21 | 14 | 5.141388 | 5 | 0.9725 | 0.24676 |

**Supplementary Table S1: Pathway enrichment analysis of unannotated median summarized features between LLC, LGG, and HBSS treated western style diet fed mice.** The table summarizes enriched pathways identified from the metabolomics dataset, including pathway name, total number of metabolites in each pathway (Pathway Total), number of detected features mapping to the pathway (Hits Total), enrichment factor, number of significant hits (Hits Sig), the expected number of hits under the null distribution (Expected), and the significance level as determined by Fisher’s exact test (*p*).

| Mebrown (HBSSvsLLC) | | | | | | |
| --- | --- | --- | --- | --- | --- | --- |
|  | Pathway total | Hits.total | Enrichment Factor | Hits.sig | Expected | P(Fisher) |
| Purine metabolism | 70 | 10 | 8.214227 | 1 | 0.12174 | 0.11656 |
| Cysteine and methionine metabolism | 33 | 7 | 7.187522 | 1 | 0.13913 | 0.13263 |
| Meblue (HBSSvsLLC) | | | | | | |
|  | Pathway total | Hits.total | Enrichment Factor | Hits.sig | Expected | P(Fisher) |
| Vitamin B6 metabolism | 9 | 1 | 20.7499 | 1 | 0.048193 | 0.048193 |
| Alanine, aspartate and glutamate metabolism | 28 | 2 | 10.37495 | 1 | 0.096386 | 0.094622 |
| Purine metabolism | 70 | 8 | 2.96428 | 1 | 0.33735 | 0.30183 |
| Metabolism of xenobiotics by cytochrome P450 | 68 | 7 | 2.593765 | 1 | 0.38554 | 0.33857 |
| Tyrosine metabolism | 42 | 10 | 1.886365 | 1 | 0.53012 | 0.44015 |
| Meturquoise (LLCvsHBSS) | | | | | | |
|  | Pathway total | Hits.total | Enrichment Factor | Hits.sig | Expected | P(Fisher) |
| Purine metabolism | 70 | 20 | 2.021369 | 7 | 3.463 | 0.034163 |
| Tryptophan metabolism | 41 | 15 | 1.764727 | 5 | 2.8333 | 0.12368 |
| Fatty acid degradation | 35 | 5 | 2.541167 | 2 | 0.78704 | 0.17156 |
| Nicotinate and nicotinamide metabolism | 15 | 5 | 2.541167 | 2 | 0.78704 | 0.17156 |
| Amino sugar and nucleotide sugar metabolism | 39 | 17 | 5.775339 | 10 | 1.7315 | 0.23175 |
| Meyellow (LLCvsHBSS) | | | | | | |
|  | Pathway total | Hits.total | Enrichment Factor | Hits.sig | Expected | P(Fisher) |
| Pentose and glucuronate interconversions | 18 | 2 | 13.33333 | 2 | 0.15 | 0.14754 |
| Steroid hormone biosynthesis | 87 | 1 | 6.666667 | 1 | 0.15 | 0.14754 |
| Selenocompound metabolism | 16 | 1 | 6.666667 | 1 | 0.15 | 0.14754 |
| Inositol phosphate metabolism | 21 | 2 | 13.33333 | 2 | 0.15 | 0.14754 |
| One carbon pool by folate | 26 | 1 | 6.666667 | 1 | 0.15 | 0.14754 |
| Megreen (HBSSvsLGGvsLLC) | | | | | | |
|  | Pathway total | Hits.total | Enrichment Factor | Hits.sig | Expected | P(Fisher) |
| Alanine, aspartate and glutamate metabolism | 28 | 2 | 13.00001 | 1 | 0.076923 | 0.074074 |
| Purine metabolism | 70 | 12 | 2.599969 | 1 | 0.38462 | 0.37037 |

**Supplementary Table S2: Pathway enrichment analysis of WGCNA modules.** The table summarizes enriched pathways identified from each module feature set as described. Table shows pathway name, total number of metabolites in each pathway (Pathway Total), number of detected features mapping to the pathway (Hits Total), enrichment factor, number of significant hits (Hits Sig), the expected number of hits under the null distribution (Expected), and the significance level as determined by Fisher’s exact test (*p*).

| **MiMeDB Restricted Pathway Enrichment Analysis (HBSS vs LLC)** | | | | | | |
| --- | --- | --- | --- | --- | --- | --- |
|  | **Pathway total** | **Hits.total** | **Enrichment Factor** | **Hits.sig** | **Expected** | **P(Fisher)** |
| **Valine, leucine and isoleucine degradation** | 35 | 11 | 4.987034 | 4 | 0.80208 | 0.030039 |
| **Butanoate metabolism** | 15 | 8 | 3.740275 | 3 | 0.80208 | 0.030039 |
| **Fatty acid degradation** | 35 | 4 | 4.363668 | 2 | 0.45833 | 0.062209 |
| **Lysine degradation** | 21 | 5 | 3.490889 | 2 | 0.57292 | 0.097145 |
| **Arginine and proline metabolism** | 35 | 13 | 2.380197 | 3 | 1.2604 | 0.1082 |
| **One carbon pool by folate** | 26 | 6 | 2.909091 | 2 | 0.6875 | 0.13654 |
| **Tryptophan metabolism** | 41 | 7 | 2.493517 | 2 | 0.80208 | 0.17914 |
| **Glutathione metabolism** | 19 | 7 | 2.493517 | 2 | 0.80208 | 0.17914 |
| **Nicotinate and nicotinamide metabolism** | 15 | 2 | 4.363573 | 1 | 0.22917 | 0.21499 |
| **Purine metabolism** | 70 | 9 | 2.18181 | 2 | 0.91667 | 0.22388 |

**Supplementary Table S3: Pathway enrichment analysis of cross referenced annotated features with microbiome-associated metabolite database (MiMeDB) between LLC and HBSS treated western style diet fed mice.** The table summarizes enriched pathways identified from the 171 cross referenced features after limiting data set to only those metabolites present within the MiMeDB. Table shows pathway name, total number of metabolites in each pathway (Pathway Total), number of detected features mapping to the pathway (Hits Total), enrichment factor, number of significant hits (Hits Sig), the expected number of hits under the null distribution (Expected), and the significance level as determined by Fisher’s exact test (*p*).


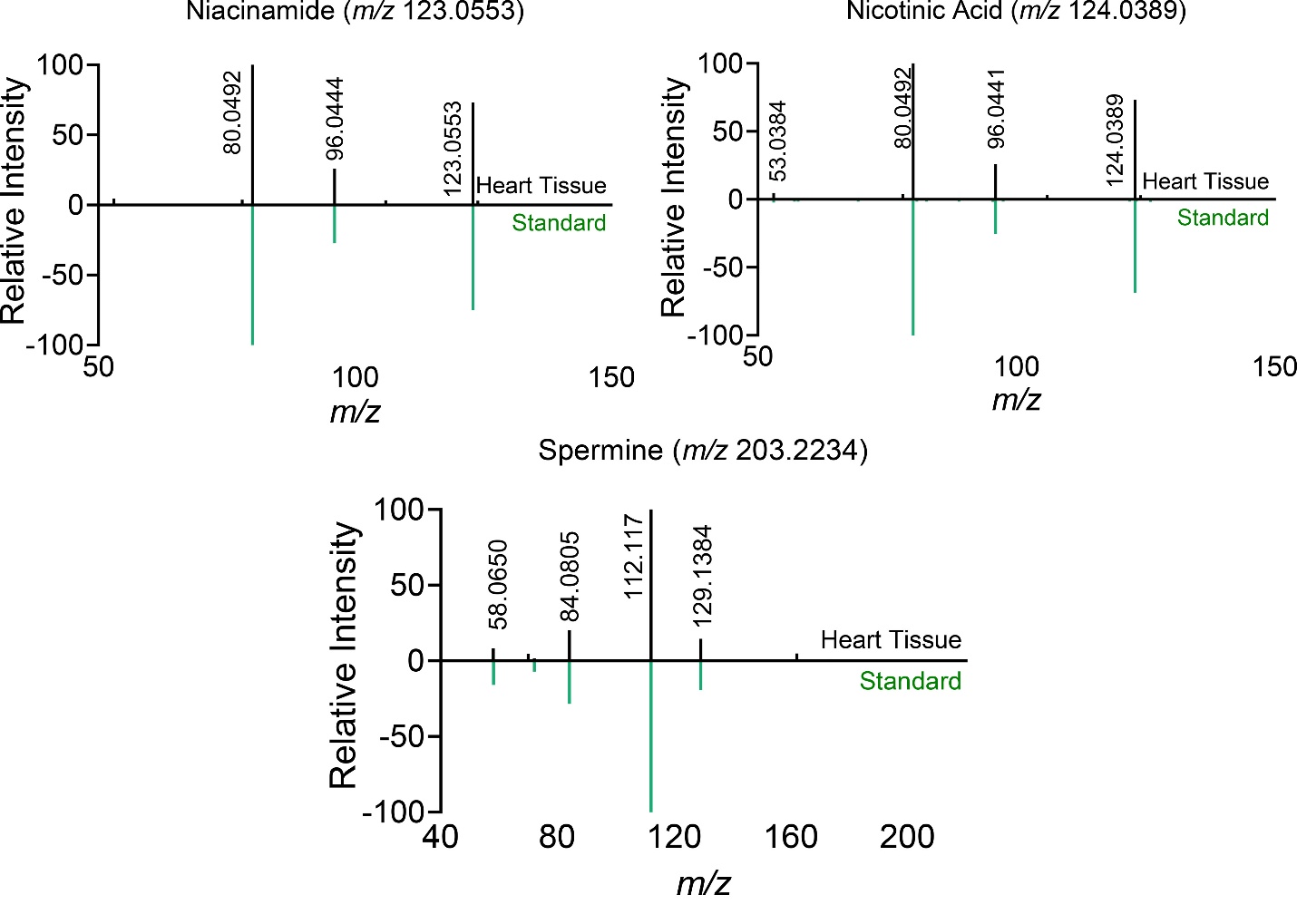


**Supplementary Figure 2. Validation of select metabolites using authenticated standards.**
Putative annotations for niacinamide, nicotinic acid, and spermine were validated using authentic standards represented by these mirror plots. Retention times were confirmed within 10 seconds of the standards. MS¹ scans were acquired at a resolution of 120,000, and MS² scans at a resolution of 60,000 using a Thermo Scientific Vanquish HPLC coupled to a Thermo Scientific Orbitrap ID-X Tribrid mass spectrometer. Ion dissociation was performed using higher-energy collisional dissociation (HCD) at a normalized collision energy of 35%.

| **MiMeDB Restricted Top Metabolites** | | | | |
| --- | --- | --- | --- | --- |
| **Name** | **Annotation**  **Confidence Score** | **LLC_vs_HBSS** (-LogP/log2FC) | **LLC_vs_LGG**  (-LogP/log2FC) | **LGG_vs_HBSS** (-LogP/log2FC) |
| (2S)-2-[5-Amino-1-(5-phospho-beta-D-ribosyl)imidazole-4-carboxamido]succinic acid | 3 | 1.406/0.803 | 0.029/0.043 | 1.052/0.76 |
| (R)-Carnitine | 3 | 0.56/0.496 | 0.403/-0.382 | 1.207/0.878 |
| 1,3,7-Trimethyluric acid | 3 | 2.297/1.339 | 1.54/0.967 | 0.559/0.372 |
| 11Z-Eicosenoic acid | 3 | 1.011/0.735 | 0.181/0.226 | 0.667/0.509 |
| 3-Methylindole | 2 | 1.72/1.007 | 0.745/0.617 | 0.404/0.39 |
| 5-Hydroxylysine | 2 | 1.034/-0.849 | 1.464/-0.922 | 0.065/0.073 |
| Acetylglycine | 2 | 0.936/-0.824 | 0.086/-0.103 | 1.182/-0.721 |
| Apigenin | 2 | 3.033/1.539 | 0.547/0.367 | 2.266/1.171 |
| Crotonoyl-CoA | 2 | 3.012/-1.375 | 0.904/-0.668 | 1.049/-0.707 |
| D-Asparagine | 3 | 0.076/-0.107 | 1.617/0.792 | 1.057/-0.899 |
| Dehydro-p-cymene | 2 | 1.43/1.128 | 0.939/0.77 | 0.747/0.358 |
| Dihydrouracil | 2 | 1.366/-0.951 | 0.032/0.034 | 1.278/-0.985 |
| Dimethylglycine | 3 | 1.502/-1.103 | 2.384/-1.024 | 0.06/-0.078 |
| Docosahexaenoic acid | 2 | 1.097/0.915 | 0.595/0.549 | 0.543/0.366 |
| Dodecenoylcarnitine | 3 | 1.151/-0.865 | 0.838/-0.612 | 0.215/-0.253 |
| FAD | 2 | 1.329/-1.098 | 0.871/-0.794 | 1.421/-0.304 |
| Flavin mononucleotide | 0 | 1.027/0.567 | 0.527/-0.534 | 1.68/1.101 |
| Formaldehyde | 0 | 1.177/-0.795 | 0.834/-0.657 | 0.106/-0.138 |
| Glutaryl-CoA | 2 | 0.294/-0.352 | 0.228/0.263 | 1.037/-0.615 |
| Glutathione | 2 | 0.145/-0.17 | 0.84/0.656 | 1.128/-0.825 |
| Hypoxanthine | 3 | 2.718/1.564 | 3.982/1.321 | 0.281/0.243 |
| Indole | 3 | 0.143/0.211 | 1.456/0.619 | 0.329/-0.408 |
| Indole-3-propionic acid | 0 | 0.96/0.785 | 1.245/0.906 | 0.126/-0.122 |
| Inosine | 3 | 3.027/1.633 | 3.538/1.185 | 0.581/0.448 |
| L-Asparagine | 3 | 0.076/-0.107 | 1.617/0.792 | 1.057/-0.899 |
| L-Dopa | 2 | 1.51/0.032 | 0.43/-0.508 | 0.464/0.539 |
| L-Glutamic acid | 3 | 0.772/0.648 | 1.324/0.851 | 0.169/-0.203 |
| L-Glutamine | 3 | 1.142/-0.878 | 0.661/-0.612 | 0.339/-0.266 |
| L-Isoleucine | 3 | 0.458/0.5 | 1.273/0.815 | 0.314/-0.315 |
| L-Methionine | 2 | 0.22/0.287 | 1.675/0.901 | 0.691/-0.615 |
| L-Proline | 2 | 0.156/0.225 | 2.238/0.818 | 0.54/-0.594 |
| L-Threonine | 2 | 0.426/0.393 | 1.009/0.853 | 0.637/-0.461 |
| Linoleic acid | 3 | 1.18/0.996 | 0.948/0.799 | 0.349/0.197 |
| LysoPC(16:0/0:0) | 3 | 1.004/-0.837 | 0.509/-0.459 | 0.433/-0.378 |
| N-Phenylacetylglycine | 2 | 0.655/0.681 | 0.129/0.178 | 2.367/0.503 |
| Niacinamide | Std Auth. | 4.363/1.944 | 2.398/1.109 | 3.706/0.835 |
| Nicotinic acid | Std Auth. | 1.194/0.995 | 0.025/-0.03 | 2.037/1.025 |
| O-Phosphoethanolamine | 3 | 0.177/-0.257 | 1.413/0.489 | 0.678/-0.746 |
| Ornithine | 3 | 0.291/0.341 | 1.11/0.704 | 0.326/-0.363 |
| PC(14:0/22:1(13Z)) | 3 | 1.032/0.784 | 1.01/0.794 | 0.008/-0.009 |
| PC(16:0/16:0) | 3 | 1.056/0.917 | 0.72/0.691 | 0.499/0.226 |
| PC(20:4(5Z,8Z,11Z,14Z)/P-18:1(11Z)) | 3 | 1.257/0.977 | 1.554/1.174 | 0.531/-0.198 |
| Pantothenic acid | 3 | 0.358/0.378 | 1.202/0.984 | 1.458/-0.606 |
| Phenylpyruvic acid | 3 | 0.161/-0.244 | 1.063/0.363 | 0.502/-0.607 |
| S-Adenosylhomocysteine | 3 | 1.516/1.148 | 1.633/1.219 | 0.291/-0.071 |
| SAICAR | 0 | 1.406/0.803 | 0.029/0.043 | 1.052/0.76 |
| Spermine | Std Auth. | 3.495/-1.679 | 0.228/0.099 | 3.562/-1.778 |
| Tartaric acid | 3 | 1.613/1.063 | 0.569/0.498 | 0.763/0.565 |
| Tetradecenoylcarnitine | 2 | 1.356/-1.117 | 1.602/-0.726 | 0.366/-0.391 |
| UDP-D-glucose | 3 | 1.024/-0.947 | 0.423/-0.255 | 0.681/-0.692 |
| Uridine | 0 | 0.058/0.074 | 1.244/1.002 | 2.943/-0.929 |
| alpha-Linolenic acid | 3 | 0.884/0.76 | 1.911/1.061 | 0.316/-0.301 |

**Supplementary Table S4. Pairwise comparison statistics for cross referenced annotated features with microbiome-associated metabolite database (MiMeDB).** This table presents all metabolites with a negative log P-value greater than 1 across pairwise comparisons. Annotation confidence scores are assigned according to the schema described by Uppal et al. (2017): Score 3 = High Confidence, 2 = Medium Confidence, 1 = Low Confidence, and 0 = MSI Level 5 (unknown). Metabolites authenticated with validated standards indicated by “Std Auth.” For each metabolite, the table includes log₂ fold changes and negative log P-values for all relevant comparisons.
